# High Prevalence of *Lactobacillus crispatus* Dominated Vaginal Microbiome Among Kenyan Secondary School Girls: Negative Effects of Poor Quality Menstrual Hygiene Management and Sexual Activity

**DOI:** 10.3389/fcimb.2021.716537

**Published:** 2021-09-21

**Authors:** Supriya D. Mehta, Garazi Zulaika, Fredrick O. Otieno, Elizabeth Nyothach, Walter Agingu, Runa Bhaumik, Stefan J. Green, Anna Maria van Eijk, Daniel Kwaro, Penelope A. Phillips-Howard

**Affiliations:** ^1^Division of Epidemiology & Biostatistics, University of Illinois at Chicago, Chicago, IL, United States; ^2^Department of Clinical Sciences, Liverpool School of Tropical Medicine, Liverpool, United Kingdom; ^3^Nyanza Reproductive Health Society, Kisumu, Kenya; ^4^Centre for Global Health Research, Kenya Medical Research Institute, Kisumu, Kenya; ^5^Department of Internal Medicine and Genomics and Microbiome Core Facility, Rush University, Chicago, IL, United States

**Keywords:** vaginal microbiome, bacterial vaginosis (BV), sexually transmitted infection (STI), menstrual health, menstrual hygiene management, adolescents and youth, Sub-Saharan Africa (SSA)

## Abstract

The vaginal microbiome (VMB) impacts numerous health outcomes, but evaluation among adolescents is limited. We characterized the VMB *via* 16S rRNA gene amplicon sequencing, and its association with Bacterial vaginosis (BV) and sexually transmitted infections (STIs; chlamydia, gonorrhea, trichomoniasis) among 436 schoolgirls in Kenya, median age 16.9 years. BV and STI prevalence was 11.2% and 9.9%, respectively, with 17.6% of girls having any reproductive tract infection. Three community state types (CST) accounted for 95% of observations: CST-I *L.crispatus*-dominant (N=178, BV 0%, STI 2.8%, sexually active 21%); CST-III *L.iners*-dominant (N=152, BV 3.3%, STI 9.7%, sexually active 35%); CST-IV *G.vaginalis*-dominant (N=83, BV 51.8%, STI 25.3%, sexually active 43%). In multivariable adjusted analyses, sexually active girls had increased odds of CST-III and CST-IV, and use of cloth to manage menses had 1.72-fold increased odds of CST-IV *vs.* CST-I. The predominance of *L.crispatus-*dominated VMB, substantially higher than observed in prior studies of young adult and adult women in sub-Saharan Africa, indicates that non-optimal VMB can be an acquired state. Interventions to maintain or re-constitute *L.crispatus* dominance should be considered even in adolescents.

## Introduction

Globally, adolescent girls and young women account for at least one-third of the 357 million curable sexually transmitted infections (STIs) occurring each year (Blum and Nelson-Mmari, 2004; Newman et al., 2015; Population Reference Bureau, 2021). STIs are syndemic with HIV. UNAIDS reports that 15% of all women living with HIV are aged 15–24 years old, 80% of whom live in sub-Saharan Africa, and 31% of new HIV infections are among adolescent girls (Amornkul et al., 2009). In western Kenya, HSV-2 increases dramatically from 10% in 13-14 year-old girls to 28% in 15-19 year-olds (Otieno et al., 2015; Kenya Ministry of Health National AIDS and STI Control Programme (NASCOP), 2020). HIV prevalence increases from 1.2% among 15-19 year-old girls to 3.4% of those aged 20-24, compared to 0.5% and 0.6% for males of the same age (Amornkul et al., 2009). Among adolescent girls, the HIV/STI epidemic overlaps with broader reproductive health concerns. For example, to attend school and obtain necessities such as sanitary products, soap and underwear, girls often engage in exchange sex (Phillips-Howard et al., 2015). Menstrual hygiene management (MHM) is a pervasive problem across low- and middle-income countries and a lack of MHM materials negatively impacts girls’ health and schooling (Sommer et al., 2016). Phillips-Howard et al. conducted a cluster randomized study of 644 girls aged 14-16 years old, comparing reusable menstrual cups to control condition of menstrual hygiene counseling (Zevin et al., 2016). After one year, menstrual cup use resulted in 35% reduction (p=0.034) in Bacterial vaginosis (BV) prevalence and 52% reduction (p=0.039) in STI prevalence compared to control condition. This decrease in STIs and BV may have been mediated by sexual practices, or the menstrual cups themselves.

Bacterial vaginosis affects 20-50% of general population women in sub-Saharan Africa (Torrone et al., 2018), and increases the risk of HIV acquisition and transmission (accounting for up to 15% of HIV infections) (Atashili et al., 2008), multiple adverse pregnancy outcomes (Eschenbach et al., 1984; Hay et al., 1994; Ralph et al., 1999), and is consistently associated with chlamydia, gonorrhea, HPV and HSV-2 (van de Wijgert et al., 2014). BV typically represents a vaginal microbiome (VMB) that is highly diverse (i.e., having many different types of bacteria) and depletion of *Lactobacillus* species (McKinnon et al., 2019). Using 16S rRNA gene amplicon sequencing, commonly occurring vaginal community state types (CST) have been identified (Ravel et al., 2011). CST-I (*Lactobacillus crispatus* dominated) has been considered an advantageous state, due to the demonstrated protective mechanisms of *L. crispatus* [e.g., maintaining acidic vaginal pH, inhibiting growth of pathogenic bacteria, activating immune cells, production of antibacterial substances, etc. (Lewis et al., 2017; Kovachev, 2018)], and due to the consistent protective association of *L. crispatus* against BV, HIV, HPV and other sexually transmitted infections (STIs) (van de Wijgert et al., 2014; McKinnon et al., 2019). On the other hand, CST-IV (a high diversity vaginal community that is usually depleted of lactobacilli) is considered a non-optimal vaginal microbiome, or “molecular BV” (McKinnon et al., 2019), and has been associated with epithelial barrier disruption and enhanced immune activation, even in the absence of clinical BV diagnosis (Zevin et al., 2016). The VMB in relation to menstrual hygiene practices and period characteristics (e.g., duration, cramping, flow) has not been rigorously assessed, and is especially lacking among adolescent girls.

We are currently evaluating the effect of menstrual cups on the VMB, BV, and STIs among secondary schoolgirls enrolled in a cluster randomized controlled trial in Siaya County, western Kenya (Zulaika et al., 2019). In the current analysis, we characterized the baseline VMB and factors associated with VMB composition in relation to sexual activity, MHM practices and menstrual characteristics, and presence of BV or STIs.

## Materials and Methods

This study was approved by the institutional review boards of the Kenya Medical Research Institutes (KEMRI, SERU #3215), Liverpool School of Tropical Medicine (LSTM, #15-005), and University of Illinois at Chicago (UIC, #2017-1301).

### Study Setting

This study used baseline data and biological specimens from the Cups and Community Health (CaCHe, pronounced “Cash-Ay”) study, a prospective cohort study of adolescent secondary school girls in Siaya County. The CaCHe study is nested in Cups or Cash for Girls (CCG), a large cluster randomized controlled trial assessing the impact of menstrual cups and cash transfer interventions on a composite outcome of school dropout, HIV and HSV-2 (Zulaika et al., 2019) (ClinicalTrials.gov NCT03051789). Siaya County area is positioned 400 km west of Nairobi, adjacent to Lake Victoria. Siaya County is largely rural and Bondo is the largest town with approximately 35,000 inhabitants. From the most recent Demographic and Health Survey, in Siaya County, women’s median age of first sexual intercourse is 16.6 years (versus 19.3 years for Nairobi and 18.0 years nationally), 19.1 years for first marriage (versus 22.1 years for Nairobi and 20.2 years nationally), and 18.4 years for first birth (versus 22.7 years for Nairobi and 20.3 years nationally) (Kenya National Bureau of Statistics and Kenya Ministry of Health, 2014). Among adult women surveyed, 15.1% had completed secondary education or more in Siaya County, compared to 51.4% of women in Nairobi and 26.9% nationally. The prevalence of HIV among adult women in the area was estimated at 25.3% in 2015 (Kenya Ministry of Health, 2021). In Health and Demographic Surveillance (HDSS) rounds taking place 2011 to 2016, HIV incidence between rounds among 15-24 year-old women and girls was 8.9%, compared to 3.2% among boys and men of same age (Borgdorff et al., 2018).

### Study Design and Participants 

The CCG trial is an open-label, 4-arm, school-cluster randomized controlled superiority trial. Schools were allocated into 4 arms (1:1:1:1) *via* block randomization: (1) provision of menstrual cups with training on safe cup use and care; (2) conditional cash transfer (CCT) based on >80% school attendance in previous term; (3) menstrual cup and CCT; and (4) usual practice. All girls received puberty and hygiene education.

For the CaCHe study, nested within the CCG trial, we aimed to enroll 20% of girls in the cup only and control arms of the CCG trial. Eligibility for CaCHe followed eligibility for CCG: attendance at a selected school, being a resident of the study area, provision of assent and parental/guardian consent, and girls had to report established menses (> 3 times). Girls were excluded if they declared pregnancy at baseline.

### Data Collection

Following written informed parental consent and assent from minors, participants self-completed a tablet-based survey in their language of choice (English or DhoLuo) to obtain socio-demographic information and to assess sexual and MHM practices. Study nurses and counsellors trained in research and survey administration provided assistance or conducted interviews as needed. Socio-demographic data included age and assessment of household amenities, including water source, light source, latrine type, and possession of a television. Household mobile phone possession was 98% and was not used in the analyses. A household amenity score having range 0-4 was created, with one pint each for piped water source, electricity for light source, flush toilet, and possession of a television. Ever having sexual intercourse was assessed in two questions to differentiate forced sex from willing sex, and *via* a series of questions around exchange sex (sex in exchange for money, goods, or favors).

### Sample Size 

CaCHe was designed to estimate the effect of menstrual cups on girls’ risk of BV, with an anticipated cumulative event rate of 30-40% among controls occurring over 30 months. In a design of 6 repeated measurements having AR(1) covariance structure, correlation between observations on the same subject ranging 0.25 to 0.4, and accounting for 20% loss to follow-up, group sample sizes of 220 in cup arm and 220 in control arm would achieve >80% power to detect 25% reduced prevalence of BV for the cup arm compared to control arm when BV prevalence is 30%, and 97% power when prevalence is 40% [p=0.05 two-sided test, two proportions in a repeated measures design; PASS v15 (Hintze, 2014)].

### Specimen Collection 

At baseline and each follow-up visit, girls were asked to take four self-collected vaginal swabs. The first swab obtained was for 16S rRNA gene amplicon sequencing (microbiome), the second for BV, the third for detection of *C. trachomatis* (CT) and *N. gonorrhoeae* (NG), and the fourth for detection of *T. vaginalis* (TV). Prior to vaginal swab collection, girls were given oral and graphic instruction on how to collect the swabs. Girls were instructed to insert each swab approximately 2-3 centimeters into the vaginal opening and to twirl the swab for 20 seconds. Each girl obtained her swabs in a private, enclosed area, with a nurse or female field assistant aiding girls with sample collection one on one. The nurses or research assistant handed the swabs to the girls sequentially, timing each collection for 20 seconds while girls were instructed to twirl, and then retrieving before passing the next swab. Nurses and research assistants prepared smears for BV immediately, with a lab assistant checking each slide for sufficiency after air drying. Swabs for amplicon sequencing, CT/NG, and TV were immediately placed on ice packs in coolers for transport.

### Detection of Bacterial Vaginosis, Sexually Transmitted Infections, and HIV 

Upon receipt at the lab, specimens for amplicon sequencing were placed at -80° C until shipment to Chicago for processing. Vaginal swabs for amplicon sequencing were collected using OMNIgene Vaginal kits (OMR-130; DNA Genotek^TM^). Swabs for CT/NG were shipped weekly for processing at the University of Nairobi Institute for Tropical and Infectious Diseases (UNITID). Following manufacturer protocol, vaginal swabs were tested for CT/NG using the GeneXpert (Cepheid, Sunnydale, California, US). Swabs for TV were processed immediately upon receipt using the OSOM TV antigen detection assay (Sekisui, Lexington, MA, US). Air-dried smears prepared from self-collected vaginal swabs were Gram stained and evaluated according to Nugent’s criteria within 48 hours of receipt; a score of 7-10 was defined as BV (Nugent et al., 1991). Finger-stick whole blood collected in EDTA tubes were tested for HIV according to Kenyan national guidelines (National AIDS and STI Control Programme, 2015). HIV positive girls were linked to care.

### STI and BV Treatment

CT, NG, and TV were treated following Kenyan National guidelines (National AIDS et al., 2015). Treatment of BV was with 2g of tinidazole once daily for two days. While not a specified regimen in the Kenyan national guidelines, we followed this alternative treatment recommendation as per U.S. Centers for Disease Control and Prevention (CDC, 2015), British Association for Sexual Health and HIV (BASHH, 2021), and International Union against Sexually Transmitted Infections/World Health Organization (IUSTI/WHO) (Sherrard et al., 2018), due to concerns of greater likelihood of gastrointestinal symptoms and decreased adherence with the longer duration regimens for metronidazole. While guidelines currently do not recommend treatment for asymptomatic BV, we treated all girls with Nugent score 7-10 due variability in recognition and reporting of symptoms (Lewis and Laurent, 2020), and potential benefits as reported in BASHH and IUSTI/WHO guidelines (Sherrard et al., 2018; BASHH, 2021), and due to the high proportion of girls reporting vaginal discharge (23%) overall, which did not differ by BV or STI status (**Table 1**). Treatment was documented for 48/49 (98%) of girls with BV, 27/27 (100%) with CT, 6/6 (100%) NG, and 13/14 (93%) TV.

**Table 1:** Distribution of baseline characteristics by bacterial vaginosis (BV) status and sexually transmitted infection (STI) status.

Variables^3^	Total N=436 n (%)	BV Status^1^		P-value	STI Status^2^		P-value^4^
		Positive, N=49 n (%)	Negative, N=387 n (%)		Positive, N=43 n (%)	Negative, N=393 n (%)	
***Socio-Demographics***							
Median Age in years (IQR)^5^	16.9 (16.0-17.9)	17.7 (16.5-18.6)	16.9 (16.0-17.7)	<0.001	17.7 (16.0-18.5)	16.9 (16.0-17.8)	0.013
Age in years, categories				0.024			0.021
14-15	52 (11.9)	3 (6.1)	49 (12.7)		3 (7.0)	49 (12.5)	
16	113 (25.9)	9 (18.4)	104 (26.9)		8 (16.0)	105 (26.7)	
17	121 (27.8)	11 (22.5)	110 (28.4)		7 (16.3)	114 (29.0)	
18	96 (22.0)	13 (26.5)	83 (21.5)		16 (37.2)	80 (20.4)	
19-22	54 (12.4)	13 (26.5)	41 (10.6)		9 (20.9)	45 (11.5)	
Latrine Type				0.883			0.181
Flush toilet	50 (11.6)	5 (10.4)	45 (11.8)		7 (17.1)	43 (11.0)	
Traditional pit	197 (45.7)	23 (47.9)	174 (45.4)		17 (41.5)	180 (46.2)	
Ventilated improved pit	171 (39.7)	18 (37.5)	153 (40.0)		14 (34.2)	157 (40.3)	
Bush, field, other	13 (3.0)	2 (4.2)	11 (2.9)		3 (7.3)	10 (2.6)	
Water Source				0.408			0.611
Bore hole	69 (16.1)	4 (8.5)	65 (17.0)		6 (14.6)	63 (16.2)	
Surface	255 (59.3)	33 (70.2)	222 (58.0)		23 (56.1)	232 (59.6)	
Pipe in house	23 (5.4)	2 (4.3)	21 (5.5)		1 (2.4)	22 (5.7)	
Rainwater	83 (19.3)	8 (17.0)	75 (19.6)		11 (26.8)	72 (18.5)	
Source of Light				0.258			0.469
Electricity	95 (22.0)	7 (14.6)	88 (23.0)		8 (19.5)	87 (22.3)	
Kerosene	171 (39.7)	23 (47.9)	148 (38.6)		22 (53.7)	149 (38.2)	
Tin lamp	61 (14.2)	10 (20.8)	51 (13.3)		5 (12.2)	56 (14.4)	
Solar	83 (19.3)	6 (12.5)	77 (20.1)		5 (12.2)	78 (20.0)	
Other	21 (4.9)	2 (4.2)	19 (5.0)		1 (2.4)	20 (5.1)	
Has Television in Home	104 (24.1)	8 (16.7)	96 (25.1)	0.200	8 (19.5)	96 (24.6)	0.468
Median household amenities (IQR): summed score of flush toilet, piped water, electricity, television	0 (0-1)	0 (0-1)	0 (0-1)	0.251	0 (0-1)	0 (0-1)	0.894
***Health Status***							
HIV Positive	6 (1.4)	3 (6.3)	3 (0.8)	0.020	0 (0.0)	6 (1.5)	>0.999
Reports having vaginal discharge	98 (23.0)	14 (29.2)	84 (22.2)	0.282	10 (25.0)	88 (22.8)	0.753
Reports having pain on urination	27 (6.3)	6 (12.5)	21 (5.6)	0.063	6 (15.0)	21 (5.4)	0.018
Past 6 months, been to health facility	246 (57.1)	30 (62.5)	216 (56.4)	0.421	25 (61.0)	221 (56.7)	0.596
Reported antibiotic use past 30 days	85 (20.0)	9 (18.8)	76 (20.1)	0.825	7 (17.5)	78 (20.2)	0.683
Body mass index, median (IQR)	21.6 (20.0-23.3)	22.6 (21.4-24.5)	21.4 (19.9-23.2)	<0.001	21.5 (20.0-23.6)	21.6 (20.0-23.3)	0.799
Body mass index, category				0.038			0.085
Underweight (<18)	25 (5.8)	0 (0.0)	25 (6.6)		5 (12.2)	20 (5.2)	
Normal (18-25)	350 (81.8)	37 (78.7)	313 (82.2)		29 (70.3)	321 (83.0)	
Overweight or obese (>25)^a^	53 (12.4)	10 (21.3)	43 (11.3)		7 (17.1)	46 (11.9)	
***Sexual Exposures***							
Past 6 months: Touched indecently by a man or boy	69 (16.0)	12 (25.0)	57 (14.9)	0.072	8 (19.5)	61 (15.6)	0.520
Past 6 months: Harassed for sex outside of school	175 (40.6)	20 (41.7)	155 (40.5)	0.874	15 (36.6)	160 (41.0)	0.582
Past 6 months: Harassed for sex at school	45 (10.4)	8 (16.7)	37 (9.7)	0.135	2 (5.9)	43 (11.0)	0.290
Ever had sex willingly	100 (23.2)	22 (45.8)	78 (20.4)	<0.001	23 (56.1)	77 (19.7)	<0.001
Ever forced or tricked to have sex	70 (16.2)	9 (18.8)	61 (15.9)	0.617	12 (29.3)	58 (14.9)	0.017
Any sexual exposure ever				0.001			<0.001
No sex ever	301 (69.8)	25 (52.1)	276 (72.1)		16 (39.0)	285 (73.1)	
Had sex willingly	60 (13.9)	14 (29.2)	46 (12.0)		13 (31.7)	47 (12.1)	
Forced or tricked intercourse	30 (7.0)	1 (2.1)	29 (7.6)		2 (4.9)	28 (7.2)	
Had sex willingly, and forced or tricked intercourse	40 (9.3)	8 (16.7)	32 (8.4)		10 (24.4)	30 (7.7)	
***Menstruation and Management***							
Median age in years at first period (IQR)	14 (14-15)	15 (14-15)	14 (14-15)	0.126	14 (13.5-15)	14 (14-15)	0.537
Early menarche (first period < 13 years of age)	19 (4.6)	0 (0)	19 (5.2)	0.147	6 (14.6)	13 (3.5)	0.001
Had period in the past 6 weeks	405 (94.0)	46 (95.8)	359 (93.7)	0.755	40 (97.6)	365 (93.6)	0.495
Pad used at last period	405 (94.0)	46 (95.8)	359 (93.7)	0.755	39 (95.1)	366 (93.9)	>0.999
Cloth used for part or all of last period	108 (25.1)	14 (29.2)	94 (24.5)	0.486	13 (31.7)	94 (24.4)	0.302

^1^ BV is defined as Nugent score 7-10.

^2^ STI is a composite of positive for C. trachomatis, N. gonorrhoeae, and/or T. vaginalis.

^3^ Not all cells sum to N due to missing values.

^4^ P-value by chi-square test unless otherwise noted; Fisher exact test used for categorical comparisons where any cell count was less than 5.

^5^ Wilcoxon rank sum test used for comparison of non-normally distributed continuous variables.

a “Overweight and obese” includes n=4 girls with BMI >30.

### DNA Extraction and Amplicon Sequencing and Annotation

Genomic DNA (gDNA) was used as template for PCR amplification of the V3-V4 variable region of bacterial 16S rRNA gene according to a two-stage PCR protocol using primers 341F and 806R, as described previously (Naqib et al., 2018; Mehta et al., 2020). After pooling of barcoded samples, amplicons were sequenced on an Illumina MiSeq instrument, implementing V3 chemistry (600 cycles). DNA extraction, library preparation and sequencing were performed by the Genome Research Core (GRC) at the University of Illinois at Chicago (UIC). Forward and reverse reads were merged using the software package PEAR (Zhang et al., 2014). Quality and primer trimmed sequence data were then processed using a standard bioinformatics pipeline for chimera removal, and annotation was conducted by University of Maryland Institute for Genomic Science (UMD IGS) (Holm et al., 2019). Subsequently, a biological observation matrix was generated at the lowest taxonomic level identifiable. Vaginal CST were identified in a reference dataset using nearest centroid classification (*VA*gina*L* community state typ*E N*earest Centro*I*d cl*A*ssifier; VALENCIA) as described in (France et al., 2020). Data were filtered to retain taxa that contributed at least 0.05% of the total sequence reads, resulting in retention of 26 vaginal taxa. There were 5 observations with <5,000 sequence reads which were excluded from analyses.

### Statistical Analysis

In this cross-sectional analysis, we examined two questions: (1) how the baseline VMB composition varied by whether girls were sexually active, and BV and/or STI presence; (2) how the baseline VMB composition varied by menstrual management practices and period characteristics.

Stacked bar plots summarizing taxa with highest relative abundance were created using Stata/SE 15. Alpha diversity indices were calculated at the amplicon sequence variant level using filtered data after rarefaction to a depth of 5,000 sequence per sample (*vegan*) (Gihring et al., 2012). We tested for global differences in vaginal community composition by BV and non-ulcerative STI status using analysis of similarity (ANOSIM) of the Bray Curtis resemblance matrix; ANOSIM is a non-parametric statistical test that assesses whether observations within a group are more similar to each other than to another group, in this way detecting differences between groups (Clarke, 1993). We visualized the relationship of global bacterial communities by BV and STI status using non-metric multidimensional scaling (NMDS) of bootstrapped averages of centroids with 100 replicates for each of the four groups representing outcome states (negative for both BV and STIs, positive for STI only, positive for BV only, positive for BV and STI). Bootstrapping is a resampling procedure that was used to estimate standard errors that allowed statistical inference on the differences between groups (Paliy and Shankar, 2016). ANOSIM and NMDS procedures were conducted in Primer-E, version 7, United Kingdom. We used multinomial logistic regression to quantify associations between explanatory factors (e.g., age, material used to manage menses, sexual activity) and CST, and Poisson regression with robust variance estimate (Barros and Hirakata, 2003) was used to quantify associations between explanatory factors and BV or STI. Because school was the unit of randomization and there were differences in distribution of socio-demographics, sexual activity, BV and STIs by school (**Supplementary Table 1**), we included a random effect for school in models of CST, BV, and STI. Multinomial logistic regression and Poisson regression were conducted in Stata/SE 15. Explanatory variables that were associated with outcomes at the p<0.10 level were entered in multivariable regression, and those with Wald p-value <0.05 were retained in multivariable models.

To identify specific taxa associated with Nugent BV and STIs, we used stability selection for feature selection [*stabs* package, implemented in R (Meinshausen and Bühlmann, 2010)]. In this approach, we applied ElasticNet regression to 250 randomly generated subsets of the vaginal microbiome data and used a cutoff of p<0.20 in combination with detection of a specific taxa in at least 60% of subsets. We chose ElasticNet regression as its ridge regression penalty supports inclusion of highly correlated variables while maintaining sparsity (Zou and Hastie, 2005). Prior to feature selection, data were center log ratio transformed following geometric Bayesian multiplicative prior imputation of zeros [*zCompositions* package, implemented in R (Palarea-Albaladejo and Martin-Fernandez, 2015)], to address sparsity while maintaining read depth. As a supplementary analysis, we also identified taxa that differed by BV and STI status using similarity of percentage analysis (Clarke, 1993), which determined the percent contributions of individual taxa to the Bray Curtis dissimilarity between groups (Primer-E, version 7, United Kingdom).

## Results

### Study Population

The median age of girls was 16.9 years (interquartile range 16.0 – 17.9) (**Table 1**). The median household amenities score – a summed score of flush toilet, piped water, electricity, and television – was zero. Majority of girls reported traditional pit (45.7%) for latrine, surface water as main water source (59.3%), and kerosene for lighting (39.7%), with 24.1% having a television. Many girls reported having been to a health facility in the past 6 months, with 20% (n=85) reporting antibiotic use in the past 30 days, primarily for fever (n=64) and generally in combination with other symptoms (such as respiratory or diarrhea). Nearly one-third (30.2%) of girls reported any prior sexual intercourse and, of those, 54% reported that they had been forced or tricked to have sex. Among sexually active girls, just 8.5% reported using a hormonal contraceptive for family planning (n=6 injectable, n=4 implant, n=1 pill) and this sparsity prevented evaluating associations with BV, STIs, or CST.

### Bacterial Community Composition Differed by BV and STI Status

**Figure 1:**
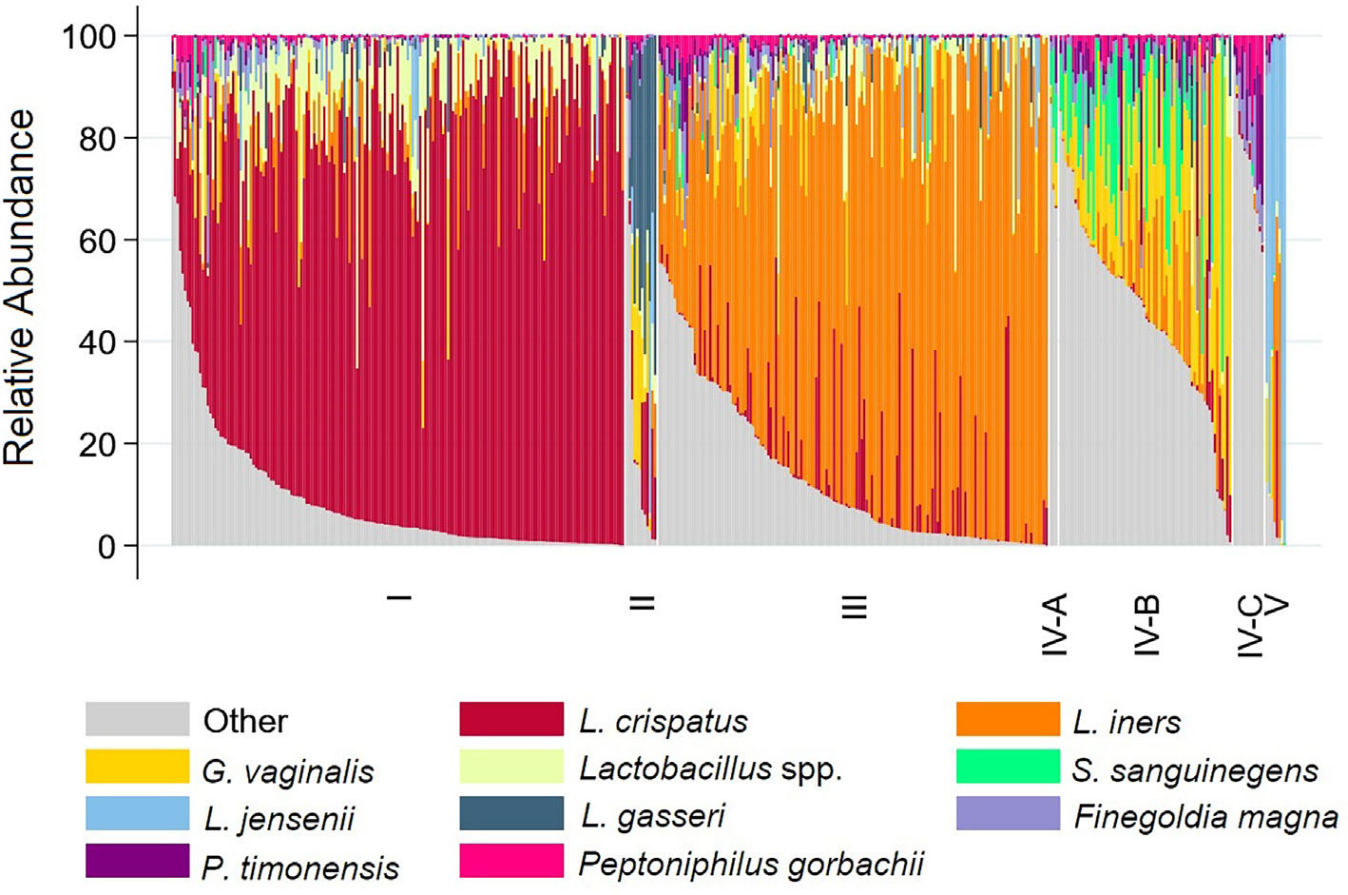
Stacked bar chart showing relative abundance of 10 taxa with highest mean relative abundance by community state type for each participant. Legend: The relative abundance of the 10 taxa with the highest mean relative abundance is shown (y-axis), with individual subjects represented by individual bars (N=431), separated by Community State Type (x-axis).

**Figure 2:**
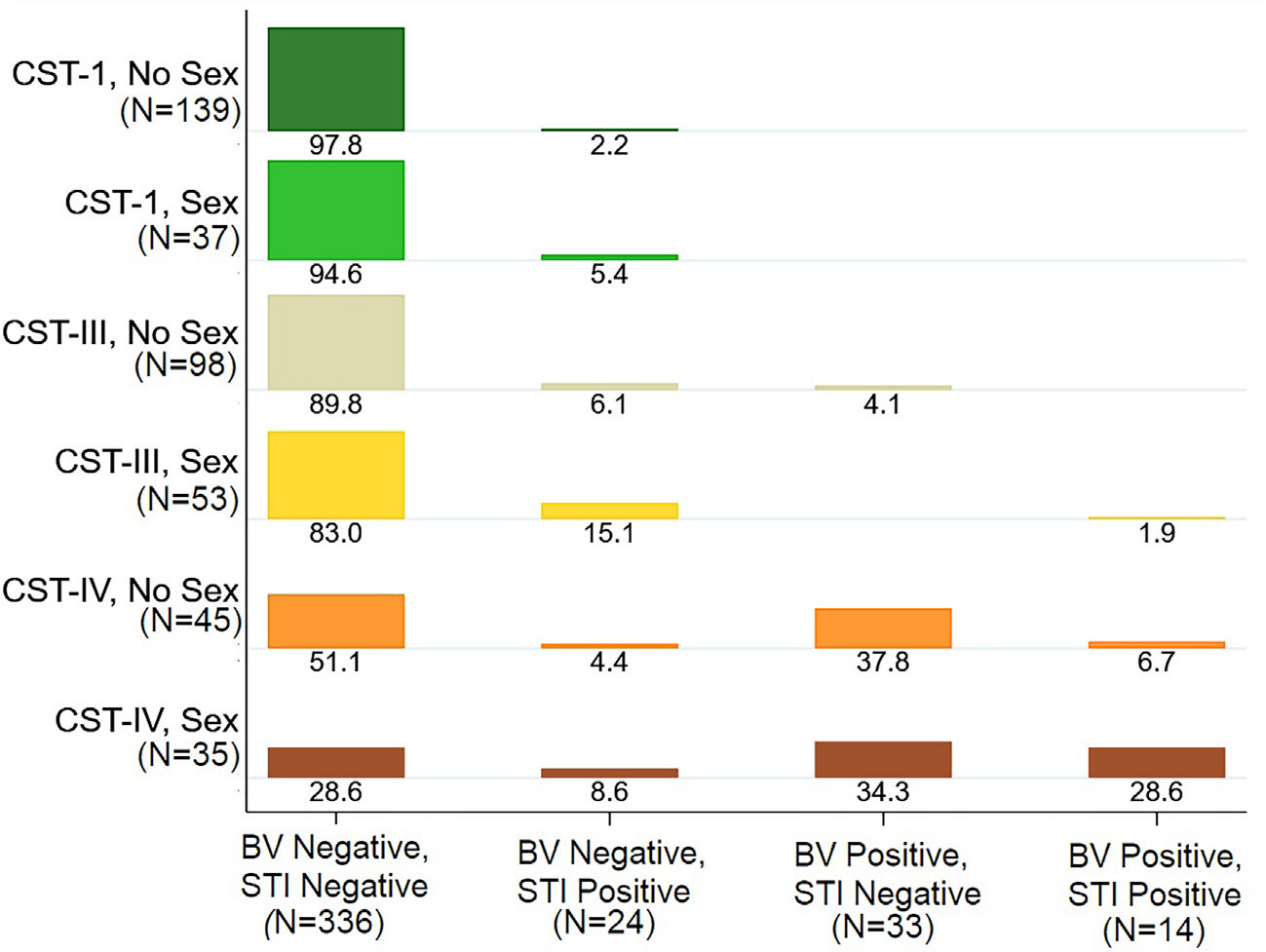
Distribution of Bacterial vaginosis (BV) and Sexually Transmitted Infection (STI) Status by Vaginal Microbiome Community State Type (CST), Stratified by Sexual Activity. Legend: The plot shows the distribution of BV and STI status by vaginal CST, stratified by sexual activity ever. For example, among girls with CST-I who do not report sexual activity, 97.8% were negative for BV and STI and 2.2% were BV Negative and STI positive. Among girls with CST-IV who reported having sexual activity, 28.6% were negative for BV and STI, 8.6% with STI only, 34.3% with BV only, and 28.6% with both BV and STI.

Three vaginal CSTs accounted for 95% of VMBs: *L. crispatus* dominant CST-I (41%), *L. iners* dominant CST-III (35%), and non-optimal CST-IV (19%) (**Table 2** and **Figure 1**). There were 12 (2.8%) girls with *L. gasseri* dominant CST-II and 8 (1.8%) girls with *L. jensenii* dominant CST-V. In keeping with the associations between non-optimal CST-IV reported in the literature (McKinnon et al., 2019), the prevalence of BV (52.4%) and non-ulcerative STIs (24.4%) was high within CST-IV, lowest in CST-I (0% BV, 2.8% non-ulcerative STI), and intermediate in CST-III (3.3% BV, 9.8% non-ulcerative STI). Overall, 59.8% of girls within CST-IV were detected with BV and/or non-ulcerative STI, compared to 2.8% within CST-I and 12.4% within CST-III (**Table 2** and **Figure 2**).

**Table 2:** Distribution of characteristics by vaginal microbiome community state type^1^.

	CST-I, N=177 *L. crispatus* dominant n (%)	CST-II, N=12 *L. gasseri* dominant n (%)	CST-III, N=153 *L iners* Dominant n (%)	CST-IV, N=81 *G. vaginalis* dominant n (%)	CST-V, N=8 *L. jensenii* dominant n (%)	^3^P-value
Bacterial Vaginosis (BV)	0 (0.0)	1 (8.3)	5 (3.3)	43 (52.4)	0 (0.0)	<0.001
Sexually Transmitted Infection (STI)	5 (2.8)	2 (16.7)	15 (9.8)	20 (24.4)	0 (0.0)	<0.001
*C. trachomatis* (CT)	4 (2.3)	2 (16.7)	8 (5.2)	12 (14.6)	0 (0.0)	0.002
*N. gonorrhoeae* (NG)	1 (0.6)	0 (0.0)	3 (2.0)	2 (2.4)	0 (0.0)	0.522
*T. vaginalis* (TV)	0 (0.0)	0 (0.0)	5 (3.3)	9 (11.0)	0 (0.0)	<0.001
BV and/or STI (CT, NG, TV)	5 (2.8)	3 (25.0)	19 (12.4)	49 (59.8)	0 (0.0)	<0.001
Ever had sex, willingly and/or forced or tricked	37 (21.0)	3 (25.0)	53 (35.1)	35 (43.8)	1 (12.5)	0.001
Median age in years (IQR)^4^	17 (16-18)	17 (16-17.8)	17 (16-18)	17 (16 – 18)	16.3 (17.5 – 18)	0.284
Median material goods point score (IQR)^4^	0 (0-1)	1 (0.25-1)	0 (0-1)	0 (0-1)	0.5 (0-1.75)	0.041
Latrine Type						0.748
Flush toilet	24 (13.6)	3 (25.0)	13 (8.6)	9 (11.3)	1 (12.5)	
Traditional pit	74 (42.1)	5 (41.7)	75 (49.7)	36 (45.0)	5 (62.5)	
Ventilated improved pit	74 (42.1)	3 (25.0)	48 (38.4)	32 (40.0)	2 (25.0)	
Bush, field, other	4 (2.3)	1 (8.3)	5 (3.3)	3 (3.8)	0 (0.0)	
Water Source						0.425
Bore hole	30 (17.1)	0 (0.0)	29 (19.2)	10 (12.7)	0 (0.0)	
Surface	103 (58.5)	8 (66.7)	84 (55.6)	52 (65.8)	4 (50.0)	
Rain water	11 (6.3)	1 (8.3)	9 (6.0)	2 (2.5)	0 (0.0)	
Pipe in house	32 (18.2)	3 (25.0)	29 (19.2)	15 (19.0)	4 (50.0)	
Source of Light						0.385
Electricity	47 (26.7)	3 (25.0)	25 (16.6)	19 (23.8)	1 (12.5)	
Kerosene	67 (38.1)	6 (50.0)	60 (39.7)	33 (41.2)	4 (50.0)	
Tin lamp	20 (11.4)	0 (0.0)	23 (15.2)	16 (20.0)	1 (12.5)	
Solar	36 (20.5)	3 (25.0)	32 (21.2)	9 (11.3)	2 (25.0)	
Other	6 (3.4)	0 (0.0)	11 (7.3)	3 (3.7)	0 (0.0)	
Has television in home	48 (27.3)	3 (25.0)	26 (17.2)	22 (27.5)	4 (50.0)	0.062
Body mass index, median (IQR)	21.6 (20.1-23.5)	21.1 (18.7-22.8)	21.2 (19.7-23.0)	22.2 (20.8-23.5)	21.1 (19.2-22.2)	0.094
Body mass index, category						0.777
Underweight (<18)	8 (4.6)	0 (0)	13 (8.7)	4 (4.9)	0 (0.0)	
Normal (18-25)	143 (82.2)	10 (83.3)	121 (80.7)	65 (80.3)	8 (100)	
Overweight or obese (>25)^2^	23 (13.2)	2 (16.7)	16 (10.7)	12 (14.8)	0 (0.0)	
School						0.346
A	52 (29.4)	2 (16.7)	36 (23.5)	21 (25.9)	4 (50.0)	
B	16 (9.0)	2 (16.7)	11 (7.2)	10 (12.4)	1 (12.5)	
C	26 (14.7)	1 (8.3)	19 (12.4)	19 (23.5)	0 (0.0)	
D	42 (23.7)	2 (16.7)	34 (22.2)	14 (17.3)	2 (25.0)	
E	20 (11.3)	2 (16.7)	25 (16.3)	11 (13.6)	1 (12.5)	
F	21 (11.9)	3 (25.0)	28 (18.3)	6 (7.4)	0 (0.0)	
Any antibiotic use past 30 days	36 (20.7)	1 (8.3)	28 (18.8)	17 (21.5)	2 (25.0)	0.847
***Menstruation & Management***						
Median age in years at first period (IQR)	14 (14-15)	14 (14-15)	14 (14-15)	14 (14-15)	15 (14-15)	0.510
Early menarche (first period < 13 years of age)	5 (3.0)	0 (0.0)	10 (6.9)	3 (3.7)	0 (0)	0.498
Cloth used to manage last menstrual period	34 (19.3)	3 (25.0)	44 (29.1)	25 (31.3)	1 (12.5)	0.143
***Period Characteristics***						
Pain or cramps last period	106 (60.2)	6 (50.0)	106 (70.2)	47 (58.8)	7 (87.5)	0.114
Menstrual bleeding last period						0.055
Light	11 (6.2)	2 (16.7)	7 (4.6)	9 (11.3)	2 (25.0)	
Normal	139 (79.0)	10 (83.3)	110 (72.9)	59 (73.7)	5 (62.5)	
Heavy	26 (15.8)	0 (0.0)	34 (22.5)	12 (15.0)	1 (12.5)	
Median days of last period (IQR)	4 (3-5)	4 (3-4.75)	4 (3-5)	4 (3-5)	4.5 (3-5.75)	0.149
1-3 days	70 (40.0)	5 (41.7)	46 (30.7)	25 (31.7)	3 (37.5)	0.341
4-6 days	93 (53.1)	5 (41.7)	92 (61.3)	43 (54.5)	5 (62.5)	
7+ days	12 (6.9)	2 (16.6)	12 (8.0)	11 (13.9)	0 (0.0)	

^1^Excludes n=4 participants with <5,000 total sequence reads.

^2^BMI category “overweight or obese” includes n=4 participants with BMI >30.

^3^P-value by chi-square test unless otherwise noted; Fisher exact test used for categorical comparisons where any cell count was less than 5.

^4^Wilcoxon rank sum test used for comparison of non-normally distributed continuous variables.

**Figure 3:**
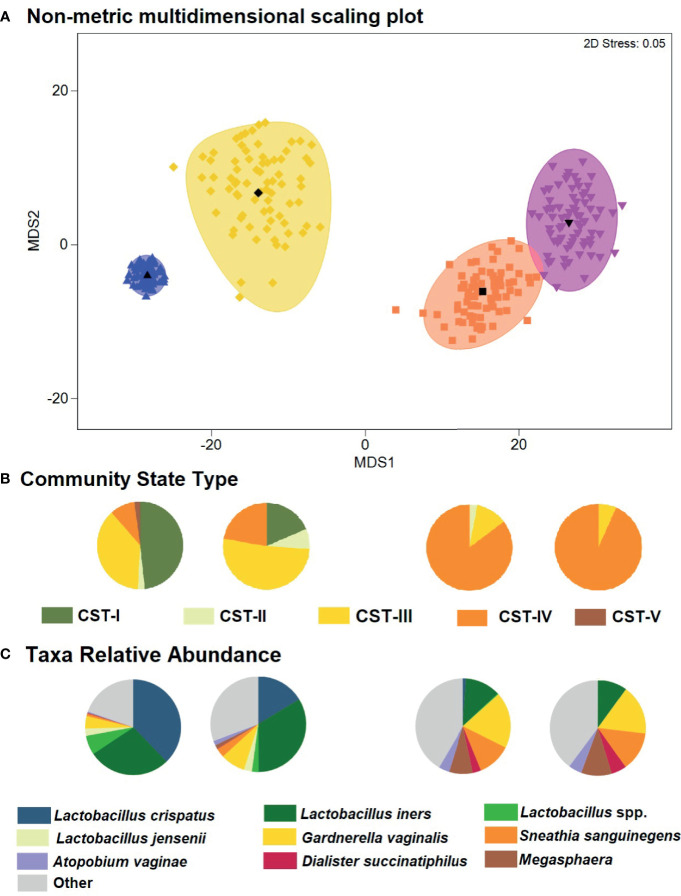
Non-metric dimensional scaling plot for each of the four outcome states for Bacterial vaginosis (BV) or sexually transmitted infection (STI) and distribution of Community State Type (CST) and taxa relative abundance. **(A)** The four different colors represent the four outcome states for Bacterial vaginosis and sexually transmitted infection. STI is a composite of infection with any of *C. trachomatis, N. gonorrhoeae, T. vaginalis.* Blue = negative for BV and all STIs; yellow = STI positive, BV negative; orange = BV positive, STI negative; pink = both STI and BV positive. Each colored mark indicates one of 100 bootstrappings of the dataset. The matching shaded area represents the 95% coverage. The black symbol at the center of each colored shape represents the average centroid of the 100 bootstraps. **(B)** Pie charts below the non-metric dimensional scaling plot show the distribution of CST, aligned to outcome states for BV and STI (N=431 individuals represented). **(C)** Pie charts below the non-metric dimensional scaling plot show the distribution of mean relative abundance of taxa identified through stability selection in association with BV and STI (N=431 individuals represented).

**Figure 4:**
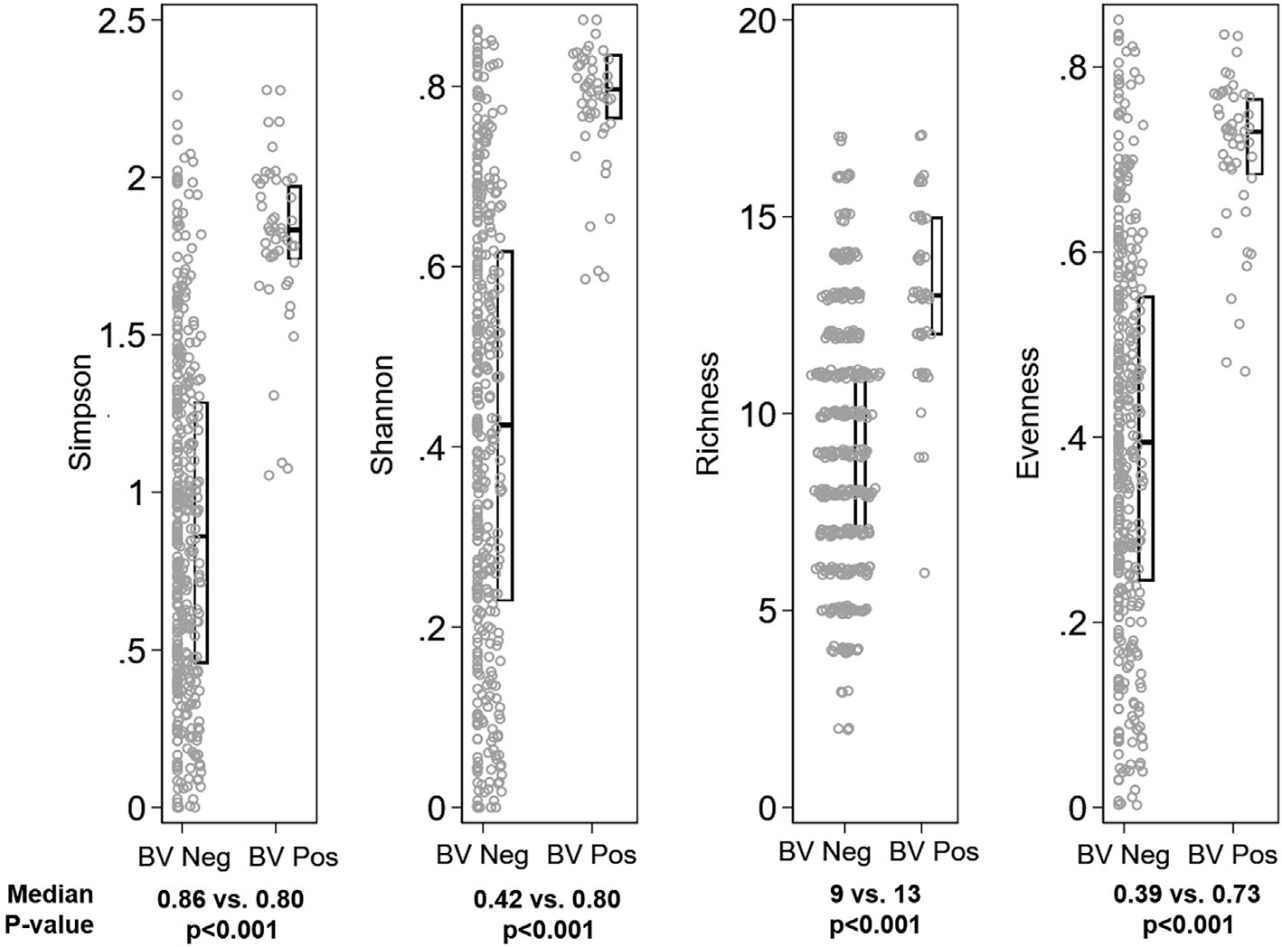
Distribution of alpha diversity metrics by Bacterial vaginosis (BV) status. Legend: The distribution of alpha diversity metrics (Simpson, Shannon, Richness, and Evenness) are shown on the y-axis, separately for girls with Nugent score 0-6 (“BV Neg”, N=382) and Nugent score 7-10 (“BV Pos”, N=49) on the x-axis. Within panels, each grey dot represents a single observation. Box plots indicate the median (horizontal bar) and interquartile range (lower 25^th^ percentile and upper 75^th^ percentile). Below each graph, the median value for each alpha diversity metric is reported for “BV Neg” and “BV Pos” observations, with Wilcoxon rank sum p-value of the comparison reported beneath the medians.

The global difference in bacterial community composition by BV and STI outcome was statistically significant (ANOSIM test, p=0.001; **Supplementary Table 2**); all pairwise comparisons were statistically significant (p=0.001, each) except for the comparison of communities in which the participant was positive for both BV and STI versus positive for BV and negative for STI (p=0.871). This difference in VMB composition is visualized in non-metric dimensional plots of the bootstrapped averages of the centroids of the four possible states of outcome (**Figure 3A**). The distribution of CST differed by BV and/or STI outcome (**Figure 3B**) and results of stability selection identified specific taxa differences between BV and STI outcomes: *L. jensenii, Dialister succinatiphilus*, *Sneathia sanguinegens, Megasphaera*, and *Lactobacillus* spp. (*Lactobacillus* identified at the genus level, but species was not identified) were associated with BV, while *Megasphaera, Atopobium vaginae, S. sanguinegens*, and *L. crispatus* were associated with STI (**Table 3** and **Figure 3C**). In supplementary analysis, the taxa contributing most to Bray Curtis dissimilarity between BV status (**Supplementary Table 3**) and STI status (**Supplementary Table 4**) were similar, with notable differences: *G. vaginalis* had strong contribution to BV positive and STI positive status, and while ElasticNet identified *L. jensenii* but not *L. crispatus* in association with BV, *L. crispatus* was the top differentiating taxa by Bray Curtis dissimilarity while *L. jensenii* was not identified as an important taxon. Girls with BV and STI had higher alpha diversity metrics (Shannon, Simpson, evenness, richness) (**Figures 4** **,** **5**), and this is in keeping with the greater frequency of diverse CST-IV among girls with BV and STIs.

**Table 3:** Results of stability selection with p=0.20 Error Bound.

Bacterial Vaginosis (BV)				Sexually Transmitted Infection (STI)			
Taxa	Proportion of Bootstrap samples Identified in	Mean Relative Abundance		Taxa	Proportion of Bootstrap samples Identified in	Mean Relative Abundance	
		BV present % (SD)	BV Absent % (SD)			STI% present (SD)	STI% absent (SD)
*Lactobacillus jensenii*	0.840	0 (0)	2.50 (10.6)	*Megasphaera*	0.612	4.43 (6.83)	0.86 (3.32)
*Sneathia sanguinegens*	0.918	12.0 (11.0)	0.96 (5.2)	*Atopobium vaginae*	0.762	2.59 (4.37)	0.63 (2.66)
*Dialister succinatiphilus*	0.920	3.55 (3.15)	0.20 (0.89)	*S. sanguinegens*	0.828	6.64 (8.45)	1.72 (6.73)
*Lactobacillus* spp.	0.996	0.19 (0.61)	6.14 (7.99)	*L. crispatus*	0.958	10.68 (24.7)	34.6 (36.9)
*Megasphaera*	0.998	8.78 (7.28)	0.25 (1.64)				

**Figure 5:**
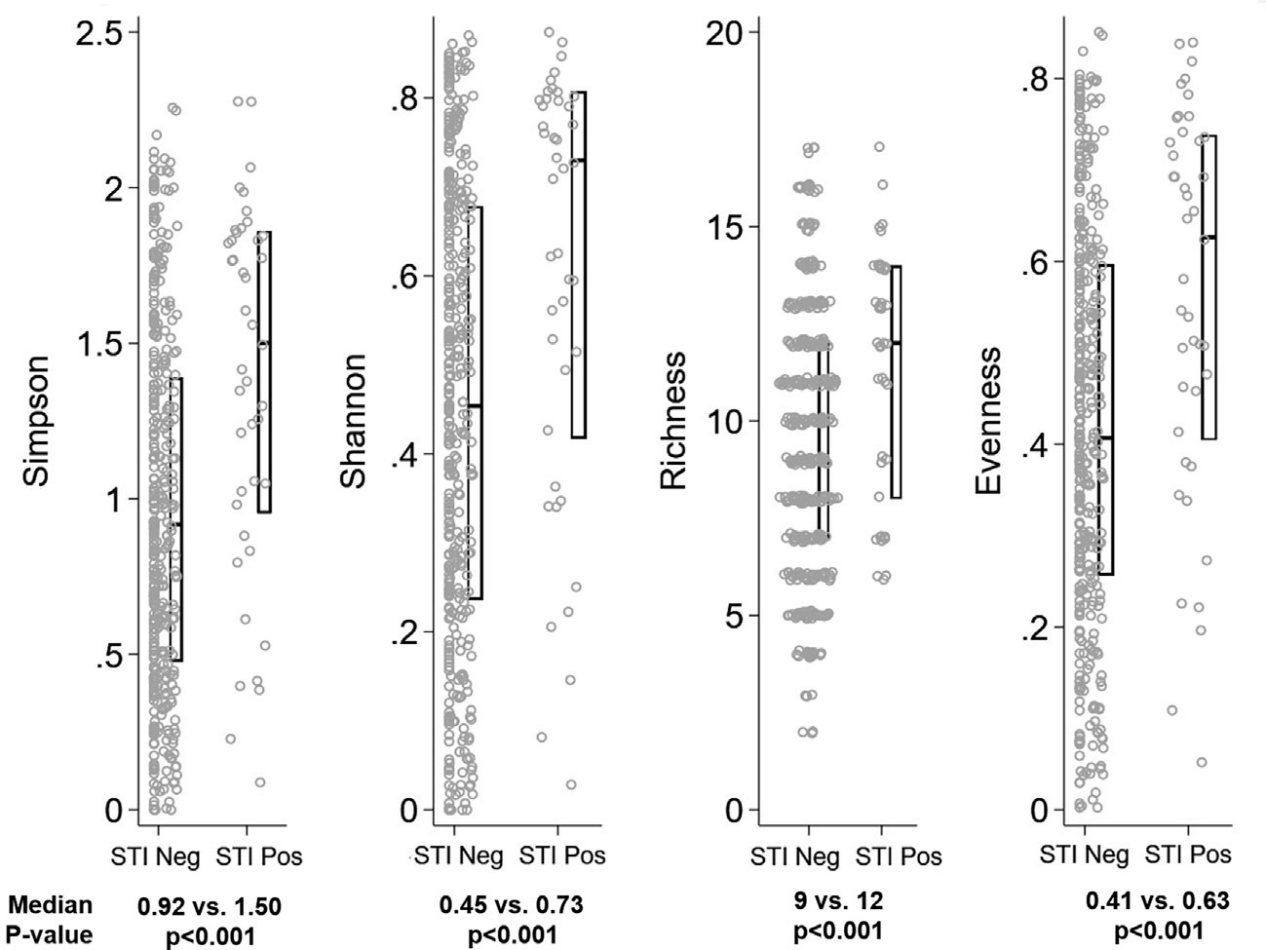
Distribution of alpha diversity metrics by Sexually Transmitted Infection (STI) status. Legend: The distribution of alpha diversity metrics (Simpson, Shannon, Richness, and Evenness) are shown on the y-axis, separately for girls testing negative for all three STIs (“STI Neg”, N=43) and testing positive for any STI (“STI Pos”, N=388) on the x-axis. Within panels, each grey dot represents a single observation. Box plots indicate the median (horizontal bar) and interquartile range (lower 25^th^ percentile and upper 75^th^ percentile). Below each graph, the median value for each alpha diversity metric is reported for “STI Neg” and “STI Pos” observations, with Wilcoxon rank sum p-value of the comparison reported beneath the medians.

### The Distribution of Community State Type Varied by Sociodemographic and Behavioral Characteristics

Household amenities scores were higher for girls with CST-II and CST-V (p=0.041), though numbers are small in these CSTs (**Table 2**). The median BMI was marginally higher for girls with CST-IV (p=0.094), in keeping with the association we observed between BMI and BV. There were no statistically significant differences in MHM or period characteristics by CST, though cloth use was more common in CST-III (29.1%) and CST-IV (31.3%) than CST-I (19.3%) (p=0.143), and when restricted to these three CSTs the difference was statistically significant (p=0.050). Notably, any sexual activity (willing or forced) was reported by 43.8% of girls with CST-IV, compared to 21% of girls with CST-I, and 35.1% of girls with CST-III. Looking at this in transpose, among girls reporting never being sexually active, 46.6% had CST-I VMB, 32.9% with CST-III, and 15.1% with CST-IV, while among girls reporting having been sexually active, 28.7% had CST-I, 41.1% CST-III, and 27.1% CST-IV (**Figure 2**).

In multinomial logistic regression (**Supplementary Table 5**) examining one covariate at a time (i.e., unadjusted), for each one year increase in age, there was a 31% increase in odds of CST-IV relative to CST-I (OR=1.31; 95% CI: 1.08 – 1.59) and increasing household amenity score was inversely associated with CST-III (OR=0.71; 95% CI: 0.55 – 0.90) relative to CST-I. Ever having been sexually active was associated with increased likelihood of CST-III (OR=2.03; 95% CI: 1.56 – 2.65) and CST-IV (OR=2.92; 95% CI: 1.26 – 6.77), as was cloth use during last period (CST-III OR=1.72; 95% CI: 1.28 – 2.31; CST-IV OR=1.90; 95% CI: 1.07 – 3.37). Increasing BMI was associated with decreasing likelihood of being in CST-III (OR=0.93; 95% CI: 0.87 – 0.99) or CST-V (OR=0.84; 95% CI: 0.71 – 0.99) relative to CST-I. In multivariable multinomial logistic regression analyses simultaneously adjusted for all variables presented (**Table 4**), all of these associations remained statistically significant (p<0.05, two sided), with no evidence of strong confounding.

**Table 4:** Results of multivariable adjusted multinomial logistic regression with random effect for school: factors associated with community state type, N=420.

	CST-II (*vs*. CST-I) OR (95% CI)	CST-III (*vs*. CST-I) OR (95% CI)	CST-IV (*vs*. CST-I) OR (95% CI)	CST-V (*vs*. CST-I) OR (95% CI)
Age in years, continuous	1.04 (0.66 – 1.63)	1.01 (0.87 – 1.17)	1.19 (1.02 – 1.38)^b^	1.26 (0.83 – 1.92)
Household amenities score, continuous	1.14 (0.73 – 1.79)	0.71 (0.56 – 0.90)^a^	0.90 (0.69 – 1.19)	1.07 (0.55 – 2.10)
Ever had sex, willingly and/or forced or tricked (*vs*. Never)	1.18 (0.19 – 7.36)	2.00 (1.63 – 2.45)^a^	2.58 (1.14 – 5.86)^b^	0.49 (0.02 – 10.1)
Cloth used during last period (*vs*. no)	1.51 (0.63 – 3.60)	1.59 (1.17 – 2.17)^a^	1.72 (1.03 – 2.86)^b^	0.72 (0.11 – 4.60)
Body mass index, continuous	0.91 (0.67-1.25)	0.93 (0.86-1.01)^c^	1.02 (0.94 – 1.11)	0.82 (0.70 – 0.98)^b^

a p-value<0.01.

b 0.01<p-value<0.05.

c 0.05<p-value<0.10.

### Prevalence of Bacterial Vaginosis and Sexually Transmitted Infections

**Figure 6:**
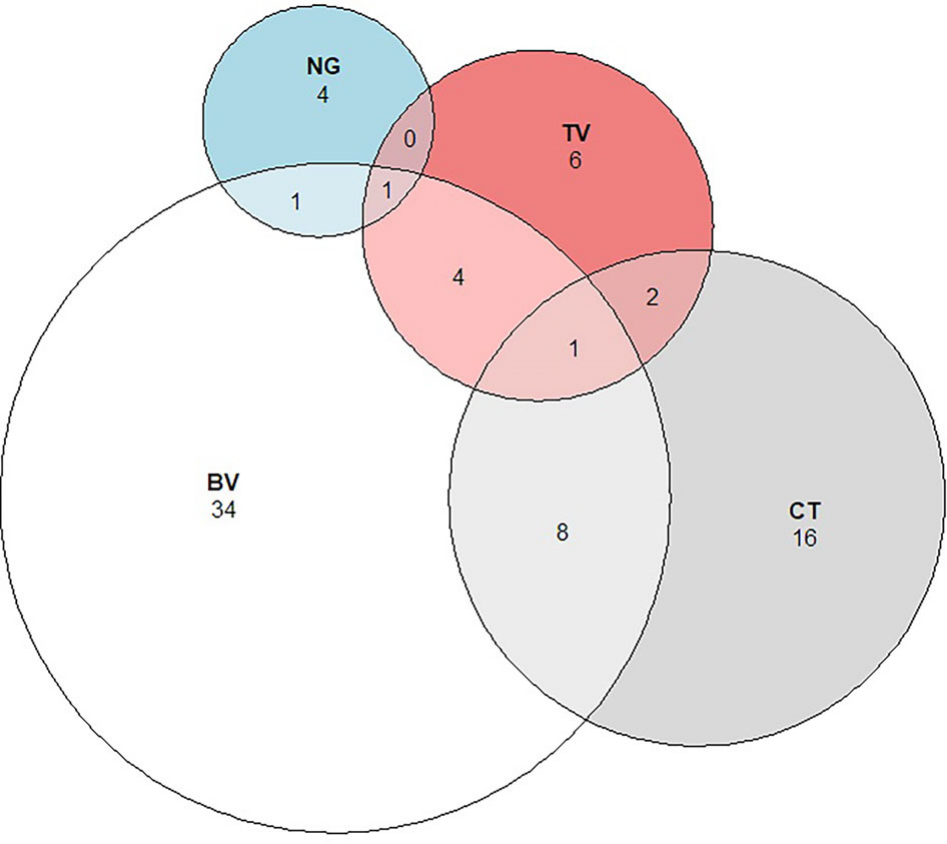
Proportional Venn diagram showing the relationship between Bacterial vaginosis and Sexually Transmitted Infections. Legend: The figure above shows the number and overlap of individuals identified with *Chlamydia trachomatis* (CT), *Neisseria gonorrhoeae* (NG), *Trichomonas vaginalis* (TV), and Bacterial vaginosis (BV).

The prevalence of STIs was 9.9% (3.0% TV, 6.2% CT, 1.4% NG), and the prevalence of BV was 11.2%. There was substantial co-infection with 31% of girls with BV having an STI, and 35% of girls with an STI also having BV (**Figure 6**).

Only two variables were associated with both BV and STI: increasing age and ever having had sex willingly (**Table 1**). Increasing BMI was associated with BV, but not with STIs. Unsurprisingly, ever reporting willing and/or forced/tricked sexual activity was more common among girls with detected BV or STI, though 52% of girls with BV and 39% of girls with STI reported never having had any type of sexual intercourse. Among girls who reported any sexual exposure, the distribution of condom use, number of lifetime partners, and age of most recent male sex partner did not differ by BV or STI status (**Supplementary Table 6**). No individual MHM practices were associated with BV or STI.

In multivariable adjusted log binomial regression analyses (**Table 5**), BV was more prevalent with increasing age (adjusted prevalence rate ratio [aPRR] = 1.24 per one year increase; 95% CI: 1.15 – 1.33), increasing BMI (aPRR = 1.13 per one unit increase; 95% CI: 1.11 – 1.15), and sexual intercourse (aPRR=2.17; 95% CI: 1.41 – 3.35), while increasing household amenities score was protective of BV (aPRR=0.76; 95% CI: 0.60 – 0.97). In a multivariable log-binomial regression model simultaneously adjusted for all variables presented, only age (aPRR=1.23; 95% CI: 1.04 – 1.46) and sexual intercourse (aPRR=3.11; 95% CI: 1.10 – 8.77) were statistically significantly associated with STI (**Table 6**). While higher BMI was associated with lower likelihood of STI, this became insignificant once adjusted for age due to the positive correlation between BMI and age.

**Table 5:** Results of poisson regression with robust variance estimate and random effect for school: crude and multivariable adjusted associations of sociodemographic and behavioral factors with bacterial vaginosis.

Variables	Crude Prevalence Rate Ratio [95% CI]	Adjusted Prevalence Rate Ratio [95% CI] N=423
Age in years, continuous	1.40 [1.26–1.55]	1.24 [1.15-1.33]
Household amenities score, continuous	0.78 [0.60-1.02]	0.76 [0.60-0.97]
Ever had sex, willingly and/or forced or tricked	2.32 [1.47-3.65]	2.17 [1.41-3.35]
Body mass index, continuous	1.15 [1.10–1.21]	1.13 [1.11–1.15]
Cloth used to manage last menstrual period	1.21 [0.78–1.86]	

Multivariable model simultaneously adjusted for all variables presented.

**Table 6:** Results of poisson regression with robust variance estimate and random effect for school: crude and multivariable adjusted associations of sociodemographic and behavioral factors with non-ulcerative sexually transmitted infection.

Variables	Crude Prevalence Rate Ratio [95% CI]	Adjusted Prevalence Rate Ratio [95% CI] N=431
Age in years, continuous	1.35 [1.12-1.61]	1.23 [1.04-1.46]
Household amenities score, continuous	0.91 [0.74-1.12]	
Ever had sex, willingly and/or forced or tricked	3.62 [1.21-10.8]	3.11 [1.10-8.77]
Early menarche (first period before age 13 years)	3.59 [1.92–6.70]	
Body mass index, category		
Underweight (<18)	ref	
Normal (18-25)	0.41 [0.23 – 0.73]	
Overweight or obese (>25)	0.66 [0.22 – 1.97]	

## Discussion

The major findings in our analyses are: (1) *L. crispatus* dominant CST-I was the most common vaginal community state type, and was more likely among girls who did not report sexual activity. (2) Girls who used cloth to manage their menses were more likely to have CST-III or non-optimal CST-IV than CST-I. (3) The prevalence of BV and STIs was high.

The majority of girls had a *L. crispatus* (41%) or *L. iners* (35%) dominant vaginal CST. This is important because numerous studies show that women of African descent are more likely to have non-optimal CST-IV vaginal community type (Ravel et al., 2011; Lewis et al., 2017). In our study of adult women (median age 23 years) in long-term sexual relationships who resided in Kisumu (approximately 70 km from Siaya County), at baseline 8.7% had *L. crispatus* dominant CST-I, 42% had *L. iners* dominant CST-III, and 47.2% had non-optimal CST-IV (Mehta et al., 2020). That such a high proportion of native Kenyan adolescent girls in our current study had *L. crispatus* dominant CST-I clearly indicates that this is a common phenotype and is most likely altered as girls become sexually active, as reflected by the increased odds of association with CST-III (aOR 2.00) and CST-IV (aOR=2.58) compared to CST-I for girls ever having had sexual exposure, adjusted for age, socioeconomic measure, and cloth use for menses. The association between older age and CST-III and CST-IV may represent that older girls have different types of sex partners, different sexual practices, or may have been sexually active longer. As the cohort is ongoing, our eventual longitudinal evaluation will be able to quantify this change over time as girls become sexually active, and among those becoming sexually active we will be able to examine the association with sexual practices and partner characteristics.

This finding has implications for the design of behavioral and biological interventions, indicating that a non-optimal VMB composition may be preventable and that adolescence could be a critical intervention point for preventing adverse reproductive health outcomes. Poor quality menstrual hygiene is modifiable, through provision of cheap accessible hygienic products instead of cloth use, and could have substantial biological consequence. Cloth use may promote non-optimal vaginal microbiome through facilitation of anaerobic bacterial growth, through improperly washed fabric (i.e., direct transfer of bacteria), or an occlusive environment. In a district level household survey of 577,758 women aged 15-49 years in India, those who used cloth during menses were more likely to report vaginal discharge in the past 3 months (aOR=1.30), adjusted for age, gynecologic factors, and socioeconomic indicators (Anand et al., 2015). Cloth use for menses has also been associated with BV among women in Tanzania (Baisley et al., 2009) and in India (Torondel et al., 2018), and tampon use has been associated with VMB composition among women in the United States (Noyes et al., 2018). Alterations in vaginal flora during menses may be modified with other MHM products such as the menstrual cup. Menstrual cups are medical grade silicone bell chambers inserted vaginally to collected menstrual flow. Among 406 U.S. women having 4,750 collective days of menstrual cup use, colonization with *Lactobacillus* was maintained at pre-cup use levels with no change in pH or colonization with *S. aureus, G. vaginalis*, or *Bacteroides* spp (North and Oldham, 2011). Systematic review with meta-analysis suggests that menstrual cups are a safe option for menstrual hygiene in low-, middle-, and high-income countries (van Eijk et al., 2019). In a cluster randomized controlled feasibility study of 644 girls aged 14-16 years old, Phillips-Howard et al. randomized girls by school cluster 1:1:1 to reusable menstrual cups, disposable sanitary pads, or standard water, sanitation and hygiene counseling (Phillips-Howard et al., 2016). The prevalence of BV (Gram stain Nugent score 7-10) was reduced by 35% (aPRR=0.65; p=0.034) for menstrual cup users (13%) compared to pad users (20%) and control subjects (19%). Menstrual cup use also resulted in 52% (p=0.039) reduction in the prevalence of STIs (composite measure of *N. gonorrhoeae*, *C. trachomatis*, *T. vaginalis*). In our current analysis, cloth use was more common among girls with BV (29.2%) than without BV (24.5%), and for girls with STI (31.7%) than without STI (24.4%), though neither difference was statistically significant. However, cloth use was significantly associated with CSTI-III (aOR=1.59) and CST-IV (aOR=1.72). While this may seem contradictory, this could reflect underreporting of cloth use, which could have attenuating effects on the measure of association with BV and STI, both having smaller sample size than CST-III and CST-IV. Of note, vaginal discharge was more commonly reported by girls using cloth (28.7%) than those without (21.1%), though not statistically significant (p=0.10; data not shown).

The prevalence of BV and STIs was high, with 9.9% of girls having STIs and 11.2% having BV. While BV is considered a sexually enhanced condition (Verstraelen et al., 2010), there are non-sexual risk factors including intravaginal and vaginal hygiene practices (Low et al., 2011), cigarette smoking (Nelson et al., 2018), and male sexual partner’s circumcision status (Liu et al., 2015). Of girls who reported ever having had sexual activity, 37% reported not knowing the male partner’s circumcision status and just 3% reported the male partner as uncircumcised [it is estimated that 40% of men in Siaya County are uncircumcised (McKinnon et al., 2019)], precluding meaningful analysis of this variable. Only one girl reported smoking cigarettes. It is a limitation that we did not ask about intravaginal practices or application of substances to the vagina as it was felt by the local study team to be too invasive and that girls would not answer due to perceived stigma. Among girls who reported they had ever had sexual activity, we did not find factors that differentiated girls with BV or STI, though this analysis was biased by underreporting of sexual activity, as evidenced by 39% of girls with STI reporting never having been sexually active. Antibiotic use within the past 30 days was common (20%), and we did not find an association between recent antibiotic use and BV, STI, or CST. This may be due to misclassification (e.g., taking anti-malarial and reporting it as antibiotic use), underreporting of antibiotics, use of antibiotics class, dose, or duration that was not strongly influential to the VMB, or because the sample represented a mixture of antibiotic classes and indications, and therefore too much noise to detect a signal.

The VMB composition differed substantially by BV and/or STI status, as demonstrated by global community comparison (ANOSIM), distribution of CSTs, and distribution of specific taxa. These differences were in keeping with previous literature. Of note, *G. vaginalis* was not identified by ElasticNet implemented within stability selection as one of the specific taxa discriminating between BV and STI states, though it is considered a key taxa in BV pathogenesis (Schwebke et al., 2014) and was one of the top taxa by contribution to Bray Curtis dissimilarity analysis. Differences in results by machine learning and ecological approaches highlight the importance of using different analytic approaches to maximize information gain and robustness.

### Limitations

There was substantial underreporting of sexual activity, as 39% of STIs occurred among girls who reported never having had sexual activity (willing or forced). Having a small number of girls infected with each STI, we analyzed STI as a composite of CT, NG, and TV; while data comparing the VMB composition by each pathogen are limited, the specific taxa associated with each pathogen may differ (Masha et al., 2019). Nevertheless, despite high co-infection of BV and STIs, we demonstrate that taxa associated with STIs differ from those associated with BV and longitudinal analyses will provide insight on the temporal occurrence of BV and/or STIs, and VMB composition and taxa in relation to specific STI pathogens. HIV prevalence at baseline was 1.4%, and while this is high given the young median age of girls, the number is small and we cannot relate HIV status to VMB in this analysis. Our results may not be generalizable to girls who are not in school. In this cross-sectional analysis of baseline data, we cannot be certain that exposures preceded outcomes.

## Conclusions

Nearly half of adolescent girls had a *L. crispatus* dominant VMB, differing substantially from studies of young adult and adult women in Kenya and other parts of sub-Saharan Africa. This indicates that non-optimal VMB may be an acquired state for many women and girls, and interventions to maintain or re-constitute *L. crispatus* dominance should be considered, with adolescence being a potentially critical point. Menstrual cups may be a potential intervention for preventing non-optimal vaginal microbiome composition associated with non-hygienic menstrual management.

## Data Availability Statement

The datasets presented in this study can be found in online repositories. The names of the repository/repositories and accession number(s) can be found below: https://www.ncbi.nlm.nih.gov/bioproject/PRJNA540529.

## Ethics Statement

This study was approved by the institutional review boards of the Kenya Medical Research Institutes (KEMRI, SERU #3215), Liverpool School of Tropical Medicine (LSTM, #15-005), and University of Illinois at Chicago (UIC, #2017-1301). Written informed consent to participate in this study was provided by the participants' legal guardian/next of kin.

## Author Contributions

SM: Obtained funding, study conceptualization and design, statistical analysis inference, data visualization, drafted manuscript. GZ: Study oversight and management to ensure integrity to protocols and integration of CaCHe within CCG trial, data management and cleaning, critical review and revision of manuscript. FO: Study oversight and management to ensure integrity to protocols of CaCHe, critical review and revision of manuscript. EN: Study oversight and management to ensure integrity to protocols of CCG trial, critical review and revision of manuscript. WA: Development, implementation, and oversight of laboratory protocols in Kenya, acquisition of data, microbiologic analyses and interpretation, critical review and revision of manuscript. RB: Design and execution of statistical analysis approaches, critical review and revision of manuscript. SG: Development and oversight of protocols for amplicon sequencing, microbiologic analyses and interpretation, critical review and revision of manuscript. AE: Data management and cleaning, critical review and revision of manuscript. DK: Study oversight and management to ensure integrity to protocols of CCG trial and regulatory integration of CaCHe, critical review and revision of manuscript. PP-H: Obtained funding, study oversight and management to ensure integrity to protocols, critical review and revision of manuscript. All authors contributed to the article and approved the submitted version.

## Funding

This study was supported by grant number R01-HD093780 (PI: Mehta) from the National Institutes of Health, Eunice Shriver National Institute of Child Health and Human Development, and the Joint Global Health Trials Initiative (UK-Medical Research Council/ Department for International Development/ Wellcome Trust/Department of Health and Social Care, grant MR/N006046/1, PI: Phillips-Howard). The funders have no role in the design of the study, the collection, analysis, and interpretation of data, or in writing the manuscript.

## Conflict of Interest

The authors declare that the research was conducted in the absence of any commercial or financial relationships that could be construed as a potential conflict of interest.

## Publisher’s Note

All claims expressed in this article are solely those of the authors and do not necessarily represent those of their affiliated organizations, or those of the publisher, the editors and the reviewers. Any product that may be evaluated in this article, or claim that may be made by its manufacturer, is not guaranteed or endorsed by the publisher.
