# Supplementary material for: High Prevalence of Lactobacillus crispatus Dominated Vaginal Microbiome Among Kenyan Secondary School Girls: Negative Effects of Poor Quality Menstrual Hygiene Management and Sexual Activity
Source: Front Cell Infect Microbiol. 2021 Sep 21;11:716537. doi: 10.3389/fcimb.2021.716537 (PMC8490761; doi:10.3389/fcimb.2021.716537)
Supplement: Supplementary file 1 [file DataSheet_1.docx]

**Supplemental Table 1. Comparison of Characteristics by School (randomization cluster).**

| School  Variables^1^ | A, N=115  n (%) | B, N=40  n (%) | C, N=65  n (%) | D, N=99  n (%) | E, N=59  n (%) | F, N=58  n (%) |
| --- | --- | --- | --- | --- | --- | --- |
| Median age in years (SD) | 16.9 (1.33) | 15.6 (0.91) | 17.2 (1.17) | 17.0 (0.99) | 16.8 (1.33) | 17.4 (1.58) |
| Water source  Bore hole  Surface  Rainwater  Pipe in house | 37 (32.5)  40 (35.1)  25 (21.9)  12 (10.5) | 1 (2.5)  33 (82.5)  4 (10.0)  2 (5.0) | 4 (6.3)  44 (68.8)  11 (17.2)  5 (7.8) | 10 (10.4)  73 (76.0)  12 (12.5)  1 (1.0) | 6 (10.2)  37 (62.7)  14 (23.7)  2 (3.4) | 11 (19.3)  28 (49.1)  17 (29.8)  1 (1.8) |
| Latrine Type  Flush toilet  Traditional pit  Ventilated improved pit  Bush, field, other | 11 (9.7)  47 (41.2)  53 (46.5)  3 (2.6) | 5 (12.5)  15 (37.5)  18 (45.0)  2 (5.0) | 7 (10.8)  32 (49.2)  23 (35.4)  3 (4.6) | 7 (7.3)  58 (60.4)  28 (29.2)  3 (3.1) | 13 (22.0)  17 (28.8)  29 (49.2)  0 (0.0) | 7 (12.3)  28 (49.1)  20 (35.1)  2 (3.5) |
| Source of Light  Electricity  Kerosene  Tin lamp  Solar  Other | 27 (23.7)  33 (29.0)  14 (12.3)  32 (28.1)  8 (7.0) | 9 (22.5)  14 (35.0)  4 (10.0)  7 (17.5)  6 (15.0) | 14 (21.5)  26 (40.0)  11 (16.9)  12 (18.5)  2 (3.1) | 13 (13.5)  49 (51.0)  15 (15.6)  15 (15.6)  4 (4.2) | 16 (27.1)  21 (35.6)  9 (15.3)  12 (20.3)  1 (1.7) | 16 (28.1)  28 (49.1)  8 (14.0)  5 (8.8)  0 (0.0) |
| Has television in home | 33 (29.0) | 7 (17.5) | 13 (20.0) | 24 (25.0) | 14 (23.7) | 13 (22.8) |
| Household amenities  0  1  2  3  4 | 67 (58.8)  20 (17.5)  19 (16.7)  7 (6.1)  1 (0.9) | 24 (60.0)  10 (25.0)  5 (12.5)  1 (2.5)  0 (0.0) | 43 (66.2)  10 (15.4)  8 (12.3)  3 (4.6)  1 (1.5) | 60 (62.5)  28 (29.2)  7 (7.3)  1 (1.0)  0 (0.0) | 31 (52.5)  15 (25.4)  9 (15.3)  4 (6.8)  0 (0.0) | 29 (50.9)  19 (33.3)  9 (15.8)  0 (0.0)  0 (0.0) |
| Body mass index, median (IQR) | 22.2 (20.6-23.8) | 20.8 (20.0-22.9) | 22.0 (20.5-23.5) | 21.2 (19.5-22.7) | 21.3 (20.0-23.6) | 20.9 (19.5-22.6) |
| Ever had sex, willingly and/or forced or tricked | 32 (28.1) | 12 (30.0) | 12 (18.5) | 26 (27.1) | 23 (39.7) | 24 (42.1) |
| Pad or cloth use at last period  Pad only, no cloth  Any cloth use | 84 (75.7)  27 (24.3) | 28 (75.7)  9 (24.3) | 45 (71.4)  18 (38.6) | 68 (73.1)  25 (26.9) | 41 (71.9)  16 (28.1) | 43 (79.6)  11 (20.4) |
| STI (composite of *T. vaginalis*, *N. gonorrhoeae*, *C. trachomatis*)  *T. vaginalis*  *N. gonorrhoeae*  *C. trachomatis* | 13 (11.3)  2 (1.7)  2 (1.7)  9 (7.8) | 3 (7.5)  0 (0.0)  1 (2.5)  2 (5.0) | 4 (6.2)  1 (1.5)  1 (1.5)  2 (3.1) | 10 (10.1)  4 (4.1)  1 (1.0)  5 (5.1) | 10 (17.0)  4 (6.8)  1 (1.7)  7 (11.9) | 3 (5.2)  2 (3.5)  0 (0.0)  1 (1.7) |
| BV (Nugent score 7-10) | 15 (13.0) | 3 (7.5) | 13 (2.0) | 10 (10.1) | 7 (11.9) | 1 (1.7) |
| BV and/or STI | 22 (19.1) | 5 (12.5) | 16 (24.6) | 17 (17.2) | 13 (22.0) | 3 (5.2) |
| HIV Positive | 0 (0.0) | 0 (0.0) | 3 (4.6) | 2 (2.0) | 1 (1.7) | 0 (0.0) |
| Community State Type^2^  CST-I  CST-II  CST-III  CST-IV  CST-V | 52 (45.2)  2 (1.7)  36 (31.3)  21 (18.3)  4 (3.5) | 16 (40.0)  2 (5.0)  11 (27.5)  10 (25.0)  1 (2.5) | 26 (40.0)  1 (1.5)  19 (29.2)  19 (29.2)  0 (0.0) | 42 (44.7)  2 (2.1)  33 (35.1)  15 (16.0)  2 (2.1) | 20 (33.9)  2 (3.4)  25 (42.4)  11 (18.6)  1 (1.7) | 21 (36.2)  3 (5.2)  28 (48.3)  6 (10.3)  0 (0.0) |
| Alpha diversity metrics, Median (SD)  Shannon  Simpson  Evenness  Richness | 0.92 (0.57)  0.48 (0.25)  0.43 (0.22)  9 (3.5) | 1.02 (0.57)  0.57 (0.25)  0.48 (0.22)  10 (3.0) | 1.28 (0.62)  0.58 (0.26)  0.51 (0.22)  11 (3.3) | 0.90 (0.58)  0.42 (0.25)  0.38 (0.22)  9 (3.2) | 0.89 (0.61)  0.46 (0.26)  0.41 (0.23)  10 (3.4) | 0.99 (0.57)  0.49 (0.25)  0.45 (0.22)  9 (3.6) |

^1^ Not all cells sum to N due to missing values.

^2^ Excludes 4 observations with less than 5,000 total sequence count.

**Supplementary Table 2. Results of analysis of similarity measures: Comparison of microbial community structure by BV and Sexually Transmitted Infection outcome.**

|  | R Statistic | P-value |
| --- | --- | --- |
| Pairwise tests |  |  |
| Negative for both vs. Positive for both BV and STI | 0.567 | 0.001 |
| Negative for both vs. Positive for BV and negative for STI | 0.425 | 0.001 |
| Negative for both vs. Positive for STI and negative for BV | 0.194 | 0.001 |
| Positive for BV and STI vs. Positive for BV only | -0.076 | 0.871 |
| Positive for BV and STI vs. Positive for STI only | 0.322 | 0.001 |
| Positive for BV only vs. Positive for STI only | 0.353 | 0.001 |
| Global test | 0.351 | 0.001 |

**Supplementary Table 3. Results of similarity of percentages analysis: Taxa contributing to 70% Bray Curtis dissimilarity between girls with and without Bacterial vaginosis**

| Taxon id: Taxa | Ave. Abundance^1^ | | Ave. Diss^2^ | Diss/  SD^3^ | Contrib%^4^ | Cum.%^5^ |
| --- | --- | --- | --- | --- | --- | --- |
|  | BV Negative | BV Positive |  |  |  |  |
| Species: *Lactobacillus crispatus* | 2.45 | 0.20 | 6.30 | 1.19 | 7.75 | 7.75 |
| Species: *Gardnerella vaginalis* | 0.77 | 2.70 | 5.73 | 1.56 | 7.05 | 14.79 |
| Species: *Lactobacillus iners* | 2.04 | 1.67 | 5.03 | 1.22 | 6.18 | 20.97 |
| Species: *Sneathia sanguinegens* | 0.17 | 2.04 | 4.91 | 1.53 | 6.03 | 27.00 |
| Genus: *Megasphaera* | 0.07 | 1.83 | 4.64 | 1.40 | 5.70 | 32.70 |
| Genus: *Lactobacillus* spp. | 1.43 | 0.11 | 3.53 | 1.28 | 4.34 | 37.04 |
| Species: *Atopobium vaginae* | 0.12 | 1.30 | 3.33 | 1.30 | 4.09 | 41.13 |
| Species: *Dialister succinatiphilus* | 0.09 | 1.24 | 2.93 | 1.51 | 3.60 | 44.74 |
| Species: *Sneathia amnii* | 0.10 | 1.16 | 2.81 | 1.00 | 3.45 | 48.18 |
| Species: *Prevotella timonensis* | 0.40 | 1.04 | 2.50 | 1.21 | 3.07 | 51.25 |
| Species: *Finegoldia magna* | 0.63 | 1.00 | 2.13 | 1.28 | 2.62 | 53.87 |
| Genus: *Prevotella* | 0.14 | 0.76 | 1.96 | 0.79 | 2.41 | 56.28 |
| Species: BV Associated Bacterium-2 (BVAB2)* | 0.02 | 0.79 | 1.90 | 0.94 | 2.33 | 58.62 |
| Species: *Peptoniphilus gorbachii* | 0.47 | 0.74 | 1.80 | 1.17 | 2.22 | 60.83 |
| Species: *Prevotella amnii* | 0.04 | 0.74 | 1.79 | 0.79 | 2.20 | 63.03 |
| Species: *Prevotella bivia* | 0.20 | 0.58 | 1.66 | 0.70 | 2.05 | 65.08 |
| Genus: *Firmicutes* | 0.28 | 0.47 | 1.54 | 0.62 | 1.89 | 66.96 |
| Species: BV Associated Bacterium-1 (BVAB1)* | 0.01 | 0.59 | 1.41 | 0.59 | 1.74 | 68.70 |
| Species: *Porphyromonas asaccharolytica* | 0.17 | 0.50 | 1.23 | 0.90 | 1.51 | 70.21 |

^1^Average abundance as presented in table is natural log transformed sequence counts averaged across subjects.

^2^Ave Diss = Average Bray Curtis dissimilarity

^3^Diss/SD = Dissimilarity divided by Standard Deviation

^4^Contrib % = Percent contribution to dissimilarity between BV negative and BV positive communities

^5^Cum % = Cumulative percent contribution to dissimilarity between BV negative and BV positive communities

*Uncultured species in the family *Lachnospiraceae*, order *Clostridialles*

**Supplementary Table 4. Results of similarity of percentages analysis: Taxa contributing to 70% Bray Curtis dissimilarity between girls with and without Sexually Transmitted Infection**

| Taxon id: Taxa | Ave. Abundance^1^ | | Ave. Diss^2^ | Diss/  SD^3^ | Contrib%^4^ | Cum.%^5^ |
| --- | --- | --- | --- | --- | --- | --- |
|  | STI Negative | STI Positive |  |  |  |  |
| Species: *Lactobacillus crispatus* | 2.35 | 0.88 | 6.88 | 1.05 | 9.16 | 9.16 |
| Species: *Lactobacillus iners* | 1.99 | 2.04 | 6.27 | 1.00 | 8.35 | 17.50 |
| Species: *Gardnerella vaginalis* | 0.90 | 1.75 | 4.63 | 1.17 | 6.16 | 23.67 |
| Genus: *Lactobacillus* | 1.36 | 0.50 | 3.73 | 1.17 | 4.96 | 28.62 |
| Species: *Sneathia sanguinegens* | 0.28 | 1.29 | 3.30 | 1.03 | 4.39 | 33.01 |
| Genus: *Megasphaera* | 0.20 | 0.93 | 2.49 | 0.78 | 3.32 | 36.33 |
| Species: *Atopobium vaginae* | 0.20 | 0.81 | 2.24 | 0.86 | 2.98 | 39.31 |
| Species: *Lactobacillus gasseri* | 0.31 | 0.49 | 2.24 | 0.55 | 2.98 | 42.29 |
| Species: *Finegoldia magna* | 0.66 | 0.83 | 2.20 | 1.27 | 2.93 | 45.22 |
| Species: *Sneathia amnii* | 0.16 | 0.78 | 2.01 | 0.76 | 2.68 | 47.90 |
| Species: *Prevotella timonensis* | 0.46 | 0.56 | 1.82 | 0.93 | 2.42 | 50.32 |
| Species: *Lactobacillus jensenii* | 0.29 | 0.28 | 1.75 | 0.44 | 2.33 | 52.65 |
| Species: *Peptoniphilus gorbachii* | 0.50 | 0.54 | 1.74 | 1.07 | 2.32 | 54.97 |
| Species: *Ureaplasma urealyticum* | 0.30 | 0.42 | 1.68 | 0.80 | 2.23 | 57.20 |
| Genus: *Prevotella* | 0.17 | 0.59 | 1.66 | 0.78 | 2.21 | 59.41 |
| Species: *Dialister succinatiphilus* | 0.18 | 0.62 | 1.65 | 0.80 | 2.19 | 61.60 |
| Species: *Prevotella amnii* | 0.08 | 0.52 | 1.33 | 0.63 | 1.77 | 63.37 |
| Bacteria, unclassified | 0.35 | 0.48 | 1.31 | 0.88 | 1.74 | 65.11 |
| Species: *Anaerococcus prevotii* | 0.40 | 0.26 | 1.28 | 0.86 | 1.70 | 66.81 |
| Genus: *Firmicutes* | 0.30 | 0.30 | 1.25 | 0.62 | 1.67 | 68.48 |
| Species: *Gemella haemolysans* | 0.06 | 0.46 | 1.20 | 0.67 | 1.60 | 70.08 |

^1^Average abundance as presented in table is natural log transformed sequence counts averaged across subjects.

^2^Ave Diss = Average Bray Curtis dissimilarity

^3^Diss/SD = Dissimilarity divided by Standard Deviation

^4^Contrib % = Percent contribution to dissimilarity between STI negative and STI positive communities

^5^Cum % = Cumulative percent contribution to dissimilarity between STI negative and STI positive communities

**Supplemental Table 5. Results of Unadjusted Multinomial Logistic Regression Modeling with Random Effect for School: Factors Associated with Community State Type.**

|  | CST-II  (vs. CST-I)  OR (95% CI) | CST-III  (vs. CST-I)  OR (95% CI) | CST-IV  (vs. CST-I)  OR (95% CI) | CST-V  (vs. CST-I)  OR (95% CI) |
| --- | --- | --- | --- | --- |
| Age in years, continuous | 1.02 (0.67 – 1.56) | 1.05 (0.91 – 1.22) | **1.31 (1.08 – 1.59)** | 1.15 (0.83 – 1.58) |
| Household amenities score, continuous | 1.11 (0.76 – 1.62) | **0.71 (0.55 – 0.90**) | 0.90 (0.64 – 1.26) | 1.01 (0.60 – 1.72) |
| Ever had sex, willingly and/or forced or tricked (vs. Never) | 1.25 (0.22 – 7.22) | **2.03 (1.56 – 2.65)** | **2.92 (1.26 – 6.77)** | 0.54 (0.04 – 7.81) |
| Cloth used during last period (vs. no) | 1.39 (0.49 – 3.92) | **1.72 (1.28 – 2.31)** | **1.90 (1.07 – 3.37)** | 0.60 (0.13 – 2.69) |
| Body mass index, continuous | 0.92 (0.68 – 1.25) | **0.93 (0.87 – 0.99)** | 1.04 (0.98 – 1.10) | **0.84 (0.71 – 0.99)** |

**Supplemental Table 6. Comparison of sexual exposure variables among those reporting any sexual exposure ever**

| Variables^3^ | **BV Status^1^** | | **P-value^4^** | **STI Status^2^** | | **P-value^4^** |
| --- | --- | --- | --- | --- | --- | --- |
|  | **Positive,**  **N=23**  **n (%)** | **Negative,**  **N=107**  **n (%)** |  | **Positive,**  **N=25**  **n (%)** | **Negative,**  **N=130**  **n (%)** |  |
| Married, cohabiting, or widowed | 3 (6.3) | 23 (6.0) | >0.999 | 3 (7.3) | 23 (5.9) | 0.727 |
| Age at first sex (n=49 missing)  <15 years  15 years  16 years  17-20 years | 2 (11.1)  4 (22.2)  7 (38.9)  5 (27.8) | 17 (27.0)  14 (22.2)  11 (17.5)  21 (33.3) | 0.250 | 6 (33.3)  2 (11.1)  7 (38.9)  3 (16.7) | 13 (20.6)  16 (25.4)  11 (17.5)  23 (36.5) | 0.077 |
| Sexually active for 3 or more years (n=49 missing) | 7 (38.9) | 12 (19.1) | 0.080 | 8 (44.4) | 11 (17.5) | 0.017 |
| Lifetime number of men or boys willingly had sex with or forced/tricked to have sex with  One  Two  Three or more | 16 (69.6)  4 (17.4)  3 (13.0) | 72 (75.8)  12 (12.6)  11 (11.6) | 0.685 | 19 (79.2)  4 (16.7)  1 (4.2) | 69 (73.4)  12 (12.8)  13 (13.8) | 0.411 |
| Any transactional sex: given gifts, money, or favors for sex | 11 (45.8) | 56 (48.3) | 0.827 | 11 (44.0) | 56 (48.7) | 0.670 |
| Current or most recent partner’s age  Younger than you  About the same age as you  Older than you by <5 years  Older than you by 5-10 years  Older than you by >10 years  Don’t know | 0 (0.0)  14 (60.9)  4 (21.7)  0 (0.0)  1 (4.4)  3 (13.0) | 1 (0.93)  59 (44.1)  20 (18.7)  3 (2.8)  1 (0.93)  23 (21.5) | 0.638 | 0 (0.0)  13 (52.0)  6 (24.0)  0 (0.0)  1 (4.0)  5 (20.0) | 1 (0.95)  60 (57.1)  19 (18.1)  3 (2.9)  1 (0.95)  21 (20.0) | 0.702 |
| You or partner ever used a condom | 20 (87.0) | 79 (73.8) | 0.280 | 20 (80.0) | 79 (75.2) | 0.616 |
| Currently using family planning  None  Pill  Injection  Implant  Abstinence | 18 (78.3)  1 (4.4)  1 (4.4)  3 (13.0)  0 (0.0) | 98 (93.3)  0 (0.0)  5 (4.8)  1 (1.0)  1 (1.0) | 0.015 | 23 (92.0)  0 (0.0)  0 (0.0)  1 (4.0)  1 (4.0) | 93 (90.3)  1 (1.0)  6 (5.8)  3 (2.9)  0 (0.0) | 0.278 |
| Pregnant now or ever in the past | 8 (16.7) | 6 (1.6) | <0.001 | 4 (9.8) | 10 (2.6) | 0.035 |

^1^ BV is defined as Nugent score 7-10

^2^ STI is a composite of positive for *C. trachomatis, N. gonorrhoeae,* and/or *T. vaginalis*.

^3^ Not all cells sum to N due to missing values.

^4^ P-value by chi-square test unless otherwise noted; Fisher exact test used for categorical comparisons where any cell count was less than 5.
