# Supplementary material for: High Prevalence of Lactobacillus crispatus Dominated Vaginal Microbiome Among Kenyan Secondary School Girls: Negative Effects of Poor Quality Menstrual Hygiene Management and Sexual Activity
Source: Front Cell Infect Microbiol. 2021 Sep 21;11:716537. doi: 10.3389/fcimb.2021.716537 (PMC8490761; doi:10.3389/fcimb.2021.716537)
Supplement: Supplementary file 2 [file DataSheet_2.docx]

**Statistical code**

1. **R code for stability selection**

library(zCompositions)

library(glmnet)

library(randomForest)

library(stabs)

library(lars)

# Bayesian imputation, relative abundance and centered log ratio transformation

female_gbm = cmultRepl(data,method="GBM")

femaleRA_gbm = female_gbm/rowSums(runa_gbm)

femaleCLR_gbm = scale(t(scale(t(log(femaleRA_gbm)),scale=FALSE)),scale=FALSE)

#Stability selection via elastic net regression based on minimum lambda values

(stab1.glmnet <- stabsel(x = x1, y = y1,fitfun = glmnet.lasso, cutoff = 0.8,B=250,PFER = 10))

lambda1.min <- cv.glmnet(x = x1, y = y1)$lambda.min

(stab1.maxCoef <- stabsel(x = x1, y = y1, fitfun = glmnet.lasso_maxCoef, args.fitfun = list(lambda = lambda1.min),B=250,cutoff = 0.8, PFER = 5))

# Plotting

par(mfrow = c(2, 1))

plot(stab1.maxCoef, main = "Lasso (glmnet; Maximum Coefficients)")

if (exists("stab.glmnet"))

  plot(stab1.glmnet, main = "Lasso (glmnet)")

# Selected features

stab1.maxCoef$selected

1. **Stata code for random effects regressions**

# Multivariable multinomial logistic regression model for outcome CST: Gllamm (generalized linear latent and mixed models) with “cst” as dependent variable. Covariates are roundage, hasstuff, eversex, anycloth, and bmi. The random effect is “school” the link and distribution are specified. Using adaptive quadrature with 8 numerical integration points and robust variance estimate. The base category is 1 (CST-I) and the exponentiated form (eform) of the coefficient is called.

gllamm cst roundage hasstuff eversex anycloth bmi, i(school) link(mlogit) fam(binom) adapt nip(8) eform basecat(1) robust

# Multivariable Poisson regression with robust variance estimate for outcome BV: Mixed effects generalized linear model with Poisson distribution and log link is specified with robust variance estimate. The random effect is “school”, and covariates are roundage, eversex, bmi, and hasstuff. The exponentiated form (eform) of the coefficient is called.

meglm bv0 roundage eversex bmi hasstuff || school: , family(poisson) link(log) eform vce(robust)

# Multivariable Poisson regression with robust variance estimate for outcome STI: Mixed effects generalized linear model with Poisson distribution and log link is specified with robust variance estimate. The random effect is “school”, and covariates are roundage and eversex. The exponentiated form (eform) of the coefficient is called.

meglm ctngtv0 roundage eversex || school: , family(poisson) link(log) vce(robust) eform
